# DNA Exit Ramps Are Revealed in the Binding Landscapes Obtained from Simulations in Helical Coordinates

**DOI:** 10.1371/journal.pcbi.1003980

**Published:** 2015-02-12

**Authors:** Ignacia Echeverria, Garegin A. Papoian

**Affiliations:** 1 Department of Chemistry and Biochemistry, University of Maryland, College Park, Maryland, United States of America; 2 Institute for Physical Science and Technology, University of Maryland, College Park, Maryland, United States of America; University of Houston, United States of America

## Abstract

DNA molecules are highly charged semi-flexible polymers that are involved in a wide variety of dynamical processes such as transcription and replication. Characterizing the binding landscapes around DNA molecules is essential to understanding the energetics and kinetics of various biological processes. We present a curvilinear coordinate system that fully takes into account the helical symmetry of a DNA segment. The latter naturally allows to characterize the spatial organization and motions of ligands tracking the minor or major grooves, in a motion reminiscent of sliding. Using this approach, we performed umbrella sampling (US) molecular dynamics (MD) simulations to calculate the three-dimensional potentials of mean force (3D-PMFs) for a Na+ cation and for methyl guanidinium, an arginine analog. The computed PMFs show that, even for small ligands, the free energy landscapes are complex. In general, energy barriers of up to ~5 kcal/mol were measured for removing the ligands from the minor groove, and of ~1.5 kcal/mol for sliding along the minor groove. We shed light on the way the minor groove geometry, defined mainly by the DNA sequence, shapes the binding landscape around DNA, providing heterogeneous environments for recognition by various ligands. For example, we identified the presence of dissociation points or “exit ramps” that naturally would terminate sliding. We discuss how our findings have important implications for understanding how proteins and ligands associate and slide along DNA.

## Author Summary

Protein-DNA and ion-DNA interactions are key for many essential biological activities such as DNA condensation, replication, transcription and repair. All these processes require DNA-binding proteins to associate tightly to their specific targets, while also being able to find these sites in an efficient way. To facilitate the search of their DNA targets, DNA-binding proteins often first interact non-specifically with DNA molecules and then slide along the DNA. In this paper, we quantitatively describe the energetics of sliding and binding of two small ligands to the DNA’s minor groove. We show that the minor groove geometry shapes the free-energy landscape surrounding the DNA molecule providing heterogeneous binding environment that can be exploited by DNA-binding proteins in this search for their specific recognition sites.

## Introduction

DNA is a charged, semi-flexible polymer, carrying two elementary negative charges per base-pair. Mobile counterions screen the inter-strand electrostatic repulsion, hence, stabilizing the double helix. Moreover, since DNA molecules are highly rigid, mobile ions also influence DNA’s mechanical properties, for example, favoring bending and substantially influencing DNA’s conformational preferences [1, 2]. The ionic atmosphere around DNA is distinctly nonhomogeneous, where different counterions preferentially associate, for example, with the DNA grooves or the strands [3–5]. The helical nature of DNA plays a critical role in determining the distribution of counterions, providing a variety of local environments. For example, the minor groove enhanced electrostatic potential provides binding sites to positively charged ligands. Furthermore, the width of the minor groove, which is highly correlated with its electrostatic potential, is a readout mechanism determined by the sequence dependent geometry of the DNA molecule [6]. The organization and dynamics of condensed ions can also give rise to complex interactions between DNA molecules, that lead, for example, to aggregation [7–10]. Despite substantial prior work on elucidating the counterionic atmosphere around DNA, the topography and roughness of the energy landscape for ligand binding to DNA are still not well understood. However, such binding landscape features are important, since they determine how binding partners, such as counterions, drugs or proteins, move around the DNA chain.

In the eukariotic cell nucleus, DNA molecules associate with a variety of counterions, proteins and other molecules. By associating with these molecules, the DNA chain condenses into organized chromatin structures. In this compact state, a twofold challenge needs to be overcome: the DNA-binding proteins need to associate tightly to their targets, while also being able to seek out the specific site in an efficient way among a myriad of non-specific decoys. Even though numerous studies have shed light on the molecular basis of specific protein-DNA complex formation, much less is known regarding the mechanisms allowing DNA-binding proteins to find their specific targets. Experimental [11–13], theoretical [14, 15] and computational [16–20] studies have suggested that some of the processes involved in the search procedure are: 1) one-dimensional sliding of the protein along DNA (intramolecular translocation), 2) direct transfer from one DNA segment to another and 3) jumping from one DNA segment by dissociation and re-association [11, 21–23]. The first process, protein sliding or translocation, corresponds to a one-dimensional diffusion process where proteins or other ligands first associate non-specifically to DNA molecules, followed by thermally induced motions along the DNA chains. For the term sliding to make sense, the protein needs to move significant distances on a DNA surface (in either direction) before dissociating. This basically necessitates relatively low free energy barriers with respect to the longitudinal motions along DNA and relatively high (or moderate) free energy barriers to prevent dissociation [14, 15]. Recent studies allowed visualization of single transcription factors sliding along extended DNA molecules, offering a step towards understanding the one dimensional translocation or diffusion search processes [22, 24, 25]. These experiments indirectly suggest that the protein’s translocation motion is coupled to the rotation along the DNA’s axis. It is believed that, while sliding, an ensemble of rapidly fluctuating nonspecific protein-DNA interactions allow the ligand to maintain continuous contact with the major groove, minor groove, or both [26, 27]. These interactions are presumably mostly electrostatic, however, as the protein finds its DNA target site, they switch to sequence specific interactions, such as hydrogen bonding and van der Waals interactions. For example, insertion of arginine residues into the narrow minor groove, where the electrostatic potential is strongly enhanced, is a widely used mode of non-specific protein-DNA recognition [28, 29].

In DNA regulatory processes, which are thought to be highly dynamic, the helical symmetry of the DNA segment plays a critical role in providing a unique physico-chemical environment for ligand binding to a specific DNA sequence. These different environments are widely exploited by proteins and other regulatory molecules in order to tightly regulate transcription and translation. Unlike most previous modeling efforts, the current study focuses on how counterions and charged residues might move along the DNA, where its local helical geometry is emphasized and fully taken into account. Our computational approach uses molecular dynamics (MD) simulations to determine the three dimensional potential of mean force (3D-PMF) of these charged ligands. For this purpose, we developed a helical coordinate system that allowed us to constrain the ligand to track the minor groove. We studied two small charged ligands known to localize to the minor groove: a Na^+^ ion and methyl-guanidinium, an arginine side chain analog. In prior works, tracking of helical paths was used as reaction coordinate [20]. However, a helical coordinate system tiling the three-dimensional space is introduced in this work for the first time, to the best of our knowledge,.

Focusing on two small ligands allowed us to achieve two goals: 1) investigate the accuracy and efficiency of 3D-PMF simulations when using the newly introduced helical coordinates, and, furthermore, 2) map out the fine-scale roughness of the binding free energy landscape of the minor grove. The latter investigation would be difficult to carry out with protein size probes, that would introduce significant disturbances into the minor groove. Fine-grained mapping of the binding free energy landscape provides important mechanistic information about the role of non-specific interactions in protein sliding [30, 31], as elaborated below. The computed free energy landscapes directly illustrate the binding sites and energetic barriers that may be encountered by mobile ligands or proteins. The helical coordinate system presented in this work makes it possible to straightforwardly compare the energetics of association and dissociation processes to those of sliding motions along the helical path. Furthermore, we determined the changes in the solvent structure at the interface between the ligand and the DNA molecule, highlighting the rapidly fluctuating nature of DNA-solvent-ligand nonspecific interactions. Overall, our simulation results shed light on the way the roughness of the free-energy landscape in the vicinity of a DNA segment modulates binding and diffusion of ligands.

## Results

**Figure 1:**
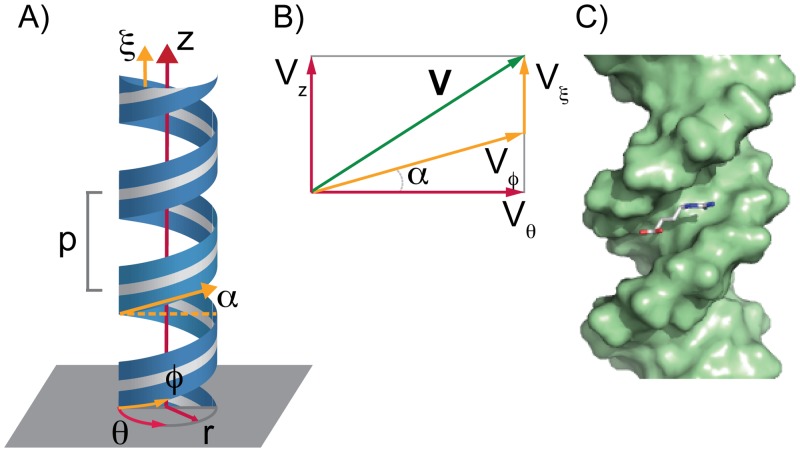
Schematic representation of the helical coordinates system. A) The helical coordinate system establishes the position of the ligand center of mass with respect to the DNA’s axis. The DNA axis was aligned to the z-axis. The helical coordinate system is defined in terms of coordinates (*ρ*, *ϕ*, *ξ*) (in yellow). Coordinates (*r*, *θ*, *z*) (in red) correspond to a cylindrical coordinate system. *p* is the pitch of the helix and *α* the pitch angle. B) The components of a vector **V** in a surface of constant *ρ* in both helical (yellow) and cylindrical coordinates (red). C) Snapshot of the initial DNA methyl-guanidinium complex.

The topological complexity of DNA molecules presents a major challenge in studying the thermodynamics and kinetics of protein-DNA and ligand-DNA interactions at the molecular level. In most cases, enhanced sampling techniques are required [16, 19, 20, 32]. Moreover, the proper choice of a reaction coordinate, which takes into account the helical symmetry of the molecule, is crucial for obtaining meaningful results that describe the energetics of processes such as ligand association or sliding. Here we present a helical coordinate system, with coordinates (*ρ*, *ϕ*, *ξ*), that uniquely defines the position of the ligands with respect to the DNA molecule. In this coordinate system, the DNA’s axis is aligned to the z-axis, such that the *ρ* and *ϕ* coordinates are equivalent in magnitude to the *r* and *θ* coordinates used in cylindrical coordinate system (*r*, *θ*, *z*) (Fig. 1). Additionally, by fixing the pitch (*p*) of the helix, this coordinate system allows to naturally track the DNA’s major or minor grooves by simple rotations. The coordinate system, by construction, decomposes the forces acting on a ligand’s center of mass in components that are: perpendicular to the DNA’s axis (**F**_*ρ*_), tangential to the helical path (**F**_*ϕ*_) and parallel to the DNA’s axis (**F**_*ξ*_) (see *Methods* section for further details). This decomposition provides the means to determine, at every point of the sampled space, the direction in which the motion of the ligand is most favorable. Importantly, all forces are conservative and uniquely defined at every point in the three dimensional space, where the latter is fully and uniquely tiled by the helical coordinate system. These properties are crucial for correctly computing the potential of mean force (PMF) for ligands binding to DNA molecules. In particular, we computed the 3D-PMF using umbrella biasing potentials (see *Methods* section and S1 Fig.) in the helical coordinate system for two small ligands: Na^+^ and methyl-guanidinium. We limited our sampling to one helical turn along the DNA’s minor grove, obtaining the binding free-energy landscape at different radial and angular positions.

### The binding free-energy landscape of Na^+^

**Figure 2:**
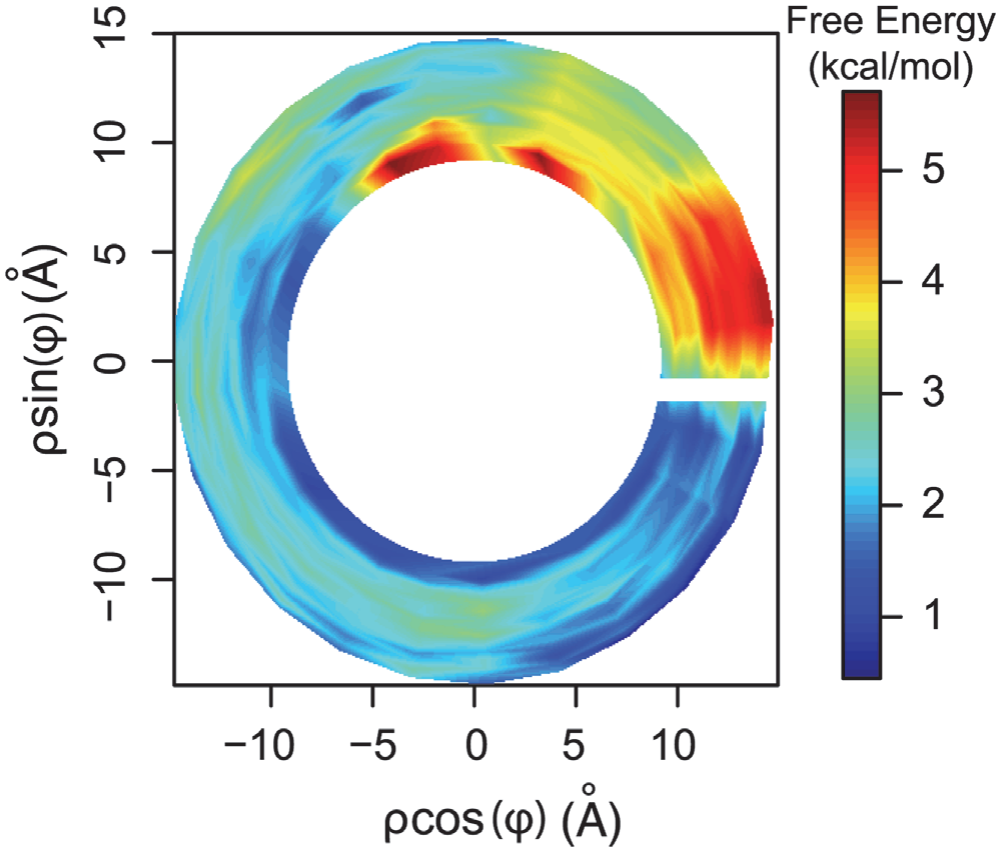
Binding free-energy landscape for Na^+^ in the minor groove: The PMF was computed for one complete turn (2*π*) along the minor groove in the helical coordinates system (see S1 Fig.). The PMF was projected to the 2D plane, such that the *ξ*-axis is perpendicular to the page (see *Methods* sections and S1 Fig.) and the obtained free-energies are those of a “ribbon” passing through the middle of the minor groove’s sampled volume. In this representation the DNA’s axis is at the center of the plot. Note that in this coordinate system there is no angular periodicity.

**Figure 3:**
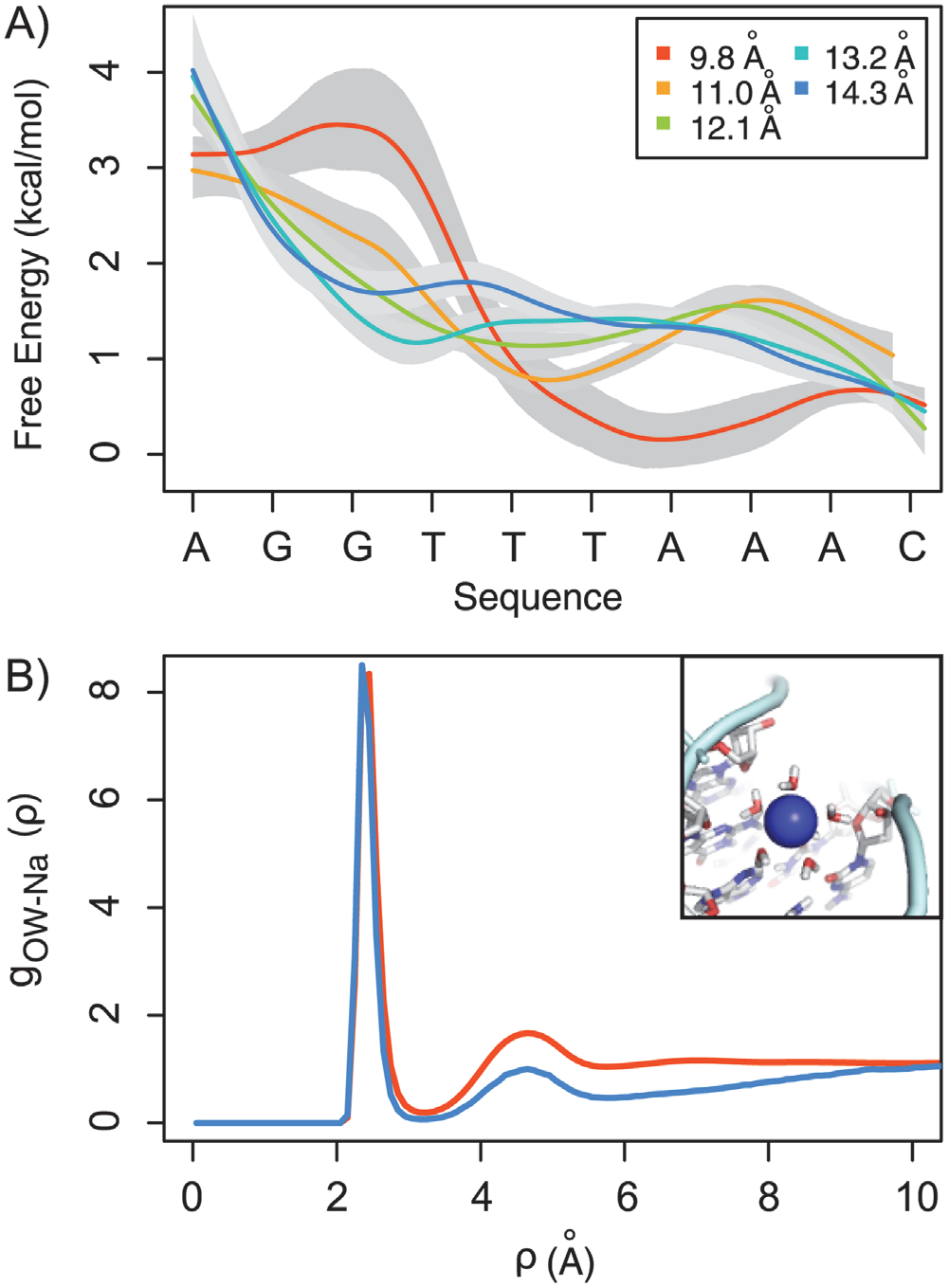
Na^+^ sliding along the minor groove. A) 1D-PMFs of one turn along the helical path at different radii from the DNA’s axis. Grey shadow correspond to one standard deviation computed using the Bootstrap method (see Methods section). B) Radial distribution functions (*g*_*OW*−*Na*^+^_) of water oxygen around the Na^+^ cations. The red line corresponds to the bulk *g*_*OW*−*Na*^+^_ and the blue line was determined considering only the Na^+^s that localized to the minor groove. Inset: snapshot from the simulations showing the localization of a hydrated Na^+^ to the minor groove.

Using all-atom umbrella sampling (US) MD simulations with the above-mentioned helical coordinate system, we calculated the PMF for a Na^+^ ion tracking the DNA’s minor groove to probe the roughness of the binding free energy landscape (Fig. 2). Notably, the free-energy landscape varies significantly along one helical turn, indicating a rough free energy surface, with large energy barriers and free energy minima. For example, in most cases, the sites of lowest free-energy are deeply buried in the minor groove (i.e. closer to the DNA’s axis). However, large free energy barriers were also identified (≳ 5 kcal/mol), possibly due to steric hindrance, as the ligands comes closer to the DNA’s axis or backbone. The large free-energy barrier in the first-quadrant (i.e. *ϕ* in the range from 0 to 90^*o*^) are due to the deformation of the DNA which causes a steric clash between the Na^+^ cation and the backbone between T5 and C6 of the 3’ to 5’ strand (S4 Fig.). On the other hand, at intermediate radii from the DNA’s axis (∼ 12 Å), the energy landscape becomes smoother (Fig. 3.A). Binding sites along the studied region are not necessarily well localized and can extend for several base pairs (Fig. 3.A). For the specific DNA sequence studied in this work, the computed energy landscape suggests that the Na^+^ ions localize to a 5 base-pair segment. Previous studies have determined that Na^+^ ions bind to AT rich regions for periods of 50 ns [3]. These binding sites are the main contributors to slowly exchanging Na^+^ ions between the DNA’s minor groove and the surrounding bulk solvent [33]. Our calculations are in agreement with these findings, but suggest that once buried into the minor groove, Na^+^ ions may easily slide along the helical path, moving slightly away from the helical axis during sliding, when needed.

### The Na^+^ is partially hydrated in the minor groove

The radial distribution function (*g*_*OW*−*Na*^+^_) of water oxygens around the Na^+^ ions (Fig. 3.B) shows that the minor groove is able to accommodate hydrated Na^+^ ions. The Na^+^ ions probed inside the minor groove maintain most of their first hydration shell (first peak, Fig. 3.B) and are characterized by partial dehydration of the second hydration shell (second peak, Fig. 3.B). This result suggests that the water molecules mediate the interactions between the Na^+^ cations and the DNA molecule and, consequently, contribute to the localization of the ions to the minor groove. This is consistent with having a shared hydration spine along the minor groove [34], where the water coordination of the Na^+^ and the DNA molecules allows the movement of the cations along the helical path, without necessarily unbinding and rebinding.

### The binding free-energy landscape of methyl-guanidinium

**Figure 4:**
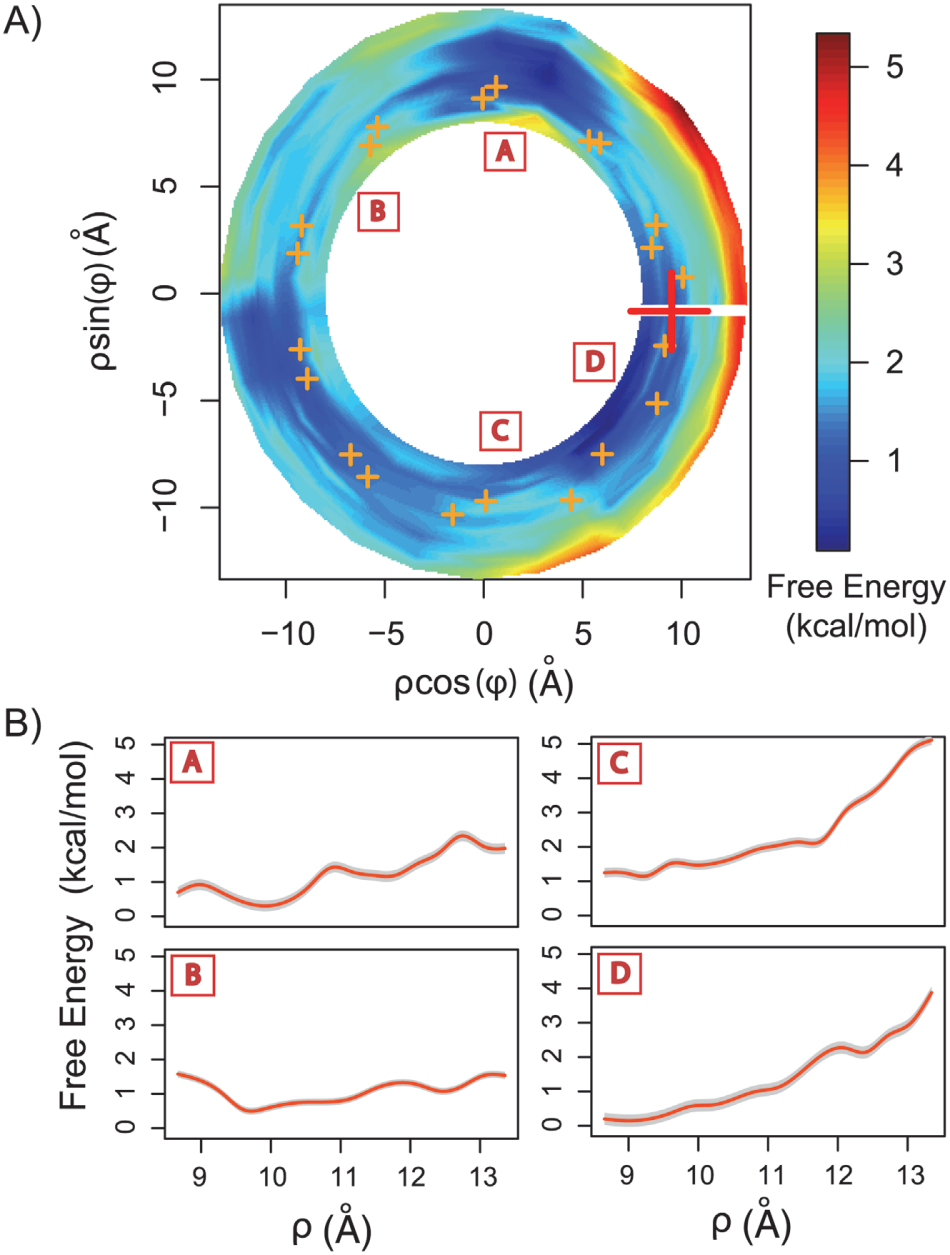
Binding free-energy landscape of methyl-guanidinium in the minor groove. A) PMF was computed for one complete turn (2*π*) along the minor groove in the helical coordinates system (see S1 Fig.). Notice that in this coordinate system there is no angular periodicity. The PMF wasprojected to the 2D plane, such that the z-axis is perpendicular to the page. Orange crosses correspond to the average position of the DNA’s backbone phosphates of the studied turn (i.e. 10.5 base pairs). The red cross correspond to the methyl-guanidinium initial center of mass position. B) 1D-PMFs of removing the methyl guanidinium from the minor groove at 4 different angular positions (red letters in boxes) as shown in A. Grey shadow correspond to one standard deviation.

**Figure 5:**
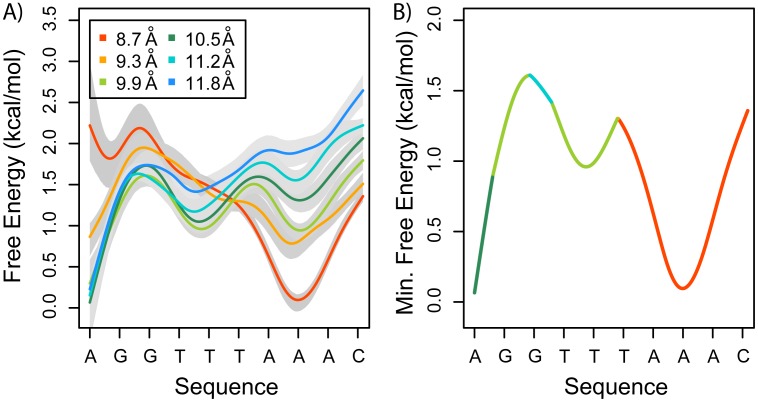
Methyl-guanidinium sliding along the minor groove. A) 1D-PMFs of one turn along the helical path at different radii from the DNA’s axis. Grey shadow correspond to one standard deviation (see Methods section). B) Minimum free energy path obtained by assuming that, at every angular position, the ligand will localize the radii of minimum free energy. The PMF has been color coded to show the radius using the same color scheme as A).

We computed the 3D-PMF for the arginine analog, methyl guanidinium, to illustrate the positional free-energy of this ligand in the minor groove. At first sight, the 2D projection of the 3D-PMF (top view, Fig. 4.A) shows that the surface of the minor groove represents an intricate landscape with various local free-energy minima, with free-energy barriers of up to 5 kcal/mol. The 1D-PMFs computed at different angular locations (Fig. 4.B) show that the free-energy barriers to removing the ligand from the minor groove can vary significantly, ranging from 1.5 to 5 kcal/mol. These free-energy differences can be interpreted as the unbinding energies at different locations. On the other hand, the free-energy profiles of the ligand sliding along the helical path (Fig. 5.A) show energy barriers ranging from 0.5 to 1.5 kcal/mol. Additionally, the minimum free energy path along the studied turn (Fig. 5.B) indicates that a small free ligand, such as methyl guanidinium, would localize to a free energy minimum having a depth of 1.5 kcal/mol. Notably, the free-energy profiles vary significantly at different radii, indicating that, at an intermediate radius (i.e. ∼ 9.3 Å) the free-energy profile for sliding has the smallest barriers (≲ 0.8 kcal/mol). Consequently, in general, the energy barriers are smaller in the angular direction than in the radial direction, making sliding the preferred mechanism for moving methyl guanidinium along the DNA chain.

**Figure 6:**
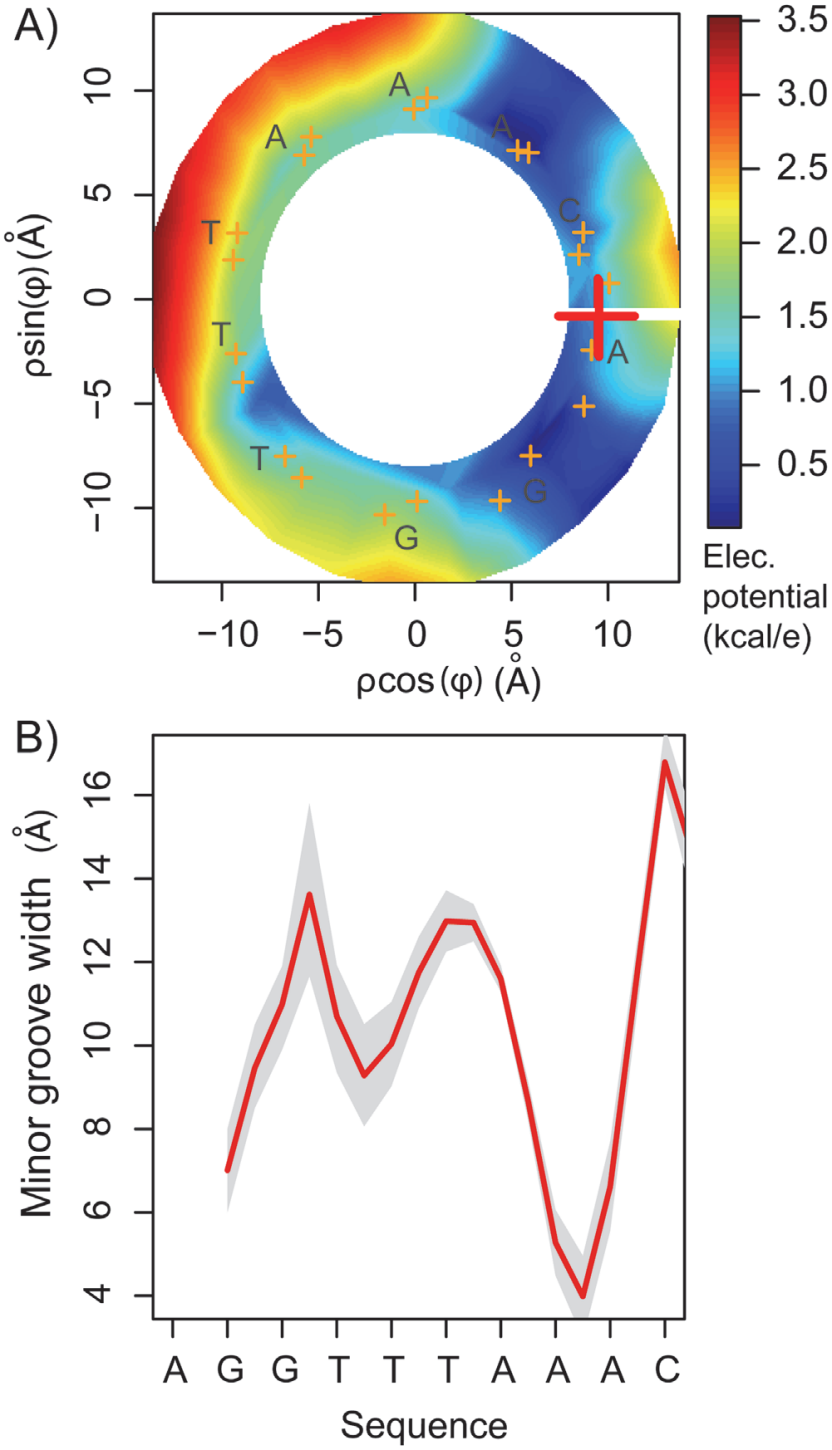
The minor groove environment. A) Electrostatic potential inside the minor groove. The potential has been rescaled such that the minima in the studied region was set to 0 to facilitate the comparison with the computed PMF. B) The minor groove width was computed using the software Curves [51]. Grey shadows correspond to one standard deviation. For examples of different structures see S5 Fig..

Fig. 6.A shows the 2D projections of the electrostatic potential inside the minor groove along one DNA turn, analogous to the 2D projections of the 3D-PMF (Fig. 5.B). The Pearson’s correlation between the free-energy (Fig. 4.A) and potential energy (Fig. 6.A) is r≃0.91. This result supports the view that the enhanced electrostatic potential in the minor groove is a key determinant of the free-energy landscape. Furthermore, the computed DNA’s minor groove width (Fig. 6.B) shows that, along the studied turn, there are two narrower segments. Qualitatively, the location and depth of the free-energy minima (Fig. 5.B) correlate with the narrow regions of the minor groove (correlation coefficient r≃0.82, Fig. 6.B) and electrostatic potentials (Fig. 6.A), in agreement with Honig and co-workers [28, 35]. However, at some different radii, the free energy profiles (Fig. 5.A) do not correlate well with the widths of the minor groove. This might have important implications for proteins binding and sliding along DNA, as further elaborated below. In addition, in the studied spatial region around the DNA segment, the ranges of the free energy differences and the electrostatic potentials differ from each other, being 5.3 kcal/mol and 3.5 kcal/mol, respectively. This range difference is most likely associated with the inaccurate treatment of complex hydration effects in the minor groove when using continuum electrostatic approaches, as well as the importance of non-electrostatic interactions. Given the high charge density of the DNA backbone, it is expected that the binding free-energy (Fig. 5) and electrostatic potential (Fig. 6) landscapes are not completely smooth at large distances (≳ 12 Å).

Our simulations indicated that the presence of the methyl-guanidinium ligand did not noticeably affect the DNA’s minor groove geometry. However, it may be possible that the timescale for groove deformations is significantly longer than the simulated time for each of our US windows.

### Methyl-guanidinium is partially hydrated in the minor groove

**Figure 7:**
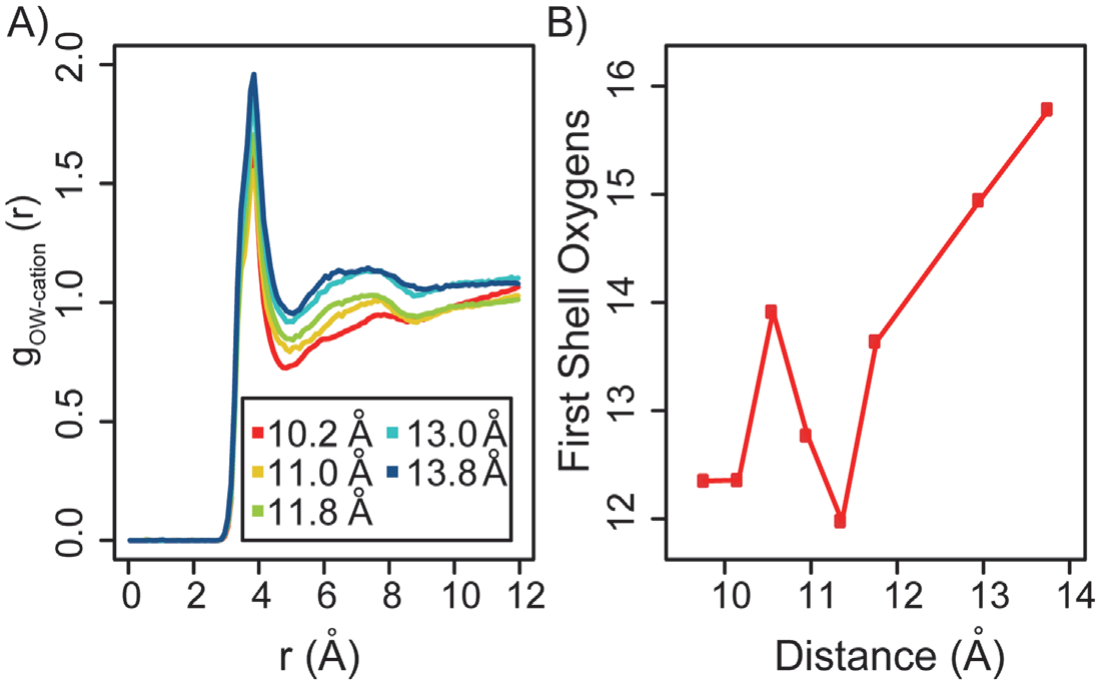
Radial distribution functions (*g*_*OW*−*cation*_) of water oxygens around the methyl-guanidinium cation. A) *g*_*OW*−*cation*_ computed at different radii from the DNA’s axis. B) Average number of water oxygen atoms 〈N〉 in the first hydration of the methyl-guanidium cation at different radius from the DNA’s axis.

As the methyl-guanidinium ligand is removed from the minor groove, the head group shows partial dehydration, as shown by the radial distribution function *g*_*OW*−*cation*_ (Fig. 7). This non-trivial hydration profile suggests that the largest dehydration occurs at an intermediate range of radii between 11 and 12 Å off the DNA’s axis (Fig. 7.B), which coincides with the position of the DNA’s phosphate groups. Consequently, the methyl-guanidinium ligands become partially dehydrated as they get into the minor groove, but once further buried into the minor groove, water molecules relocalize to the first hydration shell. Fig. 7.A also indicates partial dehydration of the second hydration shell for methyl-guanidinium cations buried in the minor groove. These hydration patterns reveal the presence of water mediated interactions [36, 37] between the methyl-guanidinium ligand and the DNA molecule. These observations suggest that the free-energy barriers of radial ligand movement not only depend on the electrostatic potential inside the minor groove but also on the sizes and the hydration levels of the ligand.

The methyl-guanidinium ligand is capable of forming direct and water-mediated hydrogen bonds with the DNA molecule. We determined that, on average, the ligand forms ∼ 0.7–1 direct hydrogen bonds with DNA, having an average life time of ∼ 25 ps. The number of hydrogen bonds decreases slightly as the ligand is removed from the minor groove, and is higher at the intermediate radii (i.e. *ρ* ∼ 9.3 − 10.0 Å). Water mediated hydrogen bonds were found to be very short lived, with an average lifetime of ∼ 2.8 ps. These results corroborate the idea that protein-DNA interactions in the minor groove are non-specific and rapidly fluctuating. In proteins, this may allow the ligand to maintain contact with the DNA molecule without the entropic cost of fixing the conformation of the side chains [27, 38].

### Implications for protein-DNA interactions

By computing the 3D-PMFs discussed above, we have characterized the physicochemical environment of the minor groove for two small charged ligands: Na^+^ and methyl-guanidinium. These maps can be used to shed light on how proteins might slide along the DNA axis while searching for their binding target. For example, the calculated PMFs show that, even for very small ligands, the surface of the minor groove is very rough and that the fine structure of the landscape varies notably at different locations. For example, smoother free energy profiles were obtained at intermediate radii (Figs. 3.A and 5.A). It is expected that small ligands, such as the studied here, will localize to the sites of lowest free energy (Fig. 5.B), even if these binding sites are buried deep inside the minor groove. However, this scenario would change for larger ligands or proteins, where the inner binding sites might not be accessible due to steric or geometrical constraints. For particular systems, such as glycosylases, experimental evidence support the concept that a single ‘wedge residue’ can be used to scan DNA [31]. In the case of sliding proteins, having a smooth free energy landscape would prove advantageous and favor moving along the helical path rather than dissociation and re-association. The methods presented here can be used to study proteins, such as transcription factors, sliding along DNA. Defining a reaction coordinate to track the minor or major grooves, or both, is natural and simple in the helical coordinate systems. Furthermore, using a coordinate system that is congruent with the geometry of DNA allows for straightforward studying of the preferred paths for proteins and other ligands.

Additionally, our work corroborates that the pre-existing geometry of the minor groove, determined mainly by the sequence, is a major determinant of the presence of binding sites for positively charged ligands. We identified that the binding sites are located in the narrow AT rich regions, where the electrostatic potential is strongly enhanced. Therefore, in the context of charged ligands bound to the DNA’s minor groove, a sliding mechanism can be described as follows: the roughness of the free energy landscape strongly depends on 1) the degree to which the ligand is buried and 2) the geometry of the minor groove. In particular, if the ligand is constrained to move at the intermediate radii, the height of the free energy barriers are comparable to the thermal fluctuations and, consequently, sliding along DNA helical path, rather than dissociation, is energetically favorable.

In the current implementation, it is required that the DNA’s axis is aligned to the z-axis, thus not being applicable in situations that require DNA translation or bending. For applications that require allowing the DNA molecules to translate or bend, our approach can be straightforwardly generalized in such a way that the helical coordinate system is defined locally, based on the instantaneous orientation of the axis of some DNA segment, while at the same time allowing for dynamic adjustment of this orientation during the simulation time course.

## Discussion

We have presented in this work a helical coordinate system, which facilitates studying the physicochemical environments surrounding DNA molecules, allowing natural tracking of DNA’s minor and major grooves. This coordinate system is fully congruent with the helical symmetry of DNA molecules and is general enough for studying the energetics of ligand or protein interactions with various DNA segments in molecular detail. As a key advantage, the helical coordinates can be used to directly obtain 3D-PMFs of ligand association to DNA, in order to determine whether sliding or unbinding is more energetically favorable at each spatial location. The 3D-PMFs can be used to determined the preferred paths of diffusive proteins and other ligands in the DNA’s vicinity.

The computed PMFs indicate that the spatially resolved binding landscapes around DNA chain segments are far from smooth, even for small ligands, showing rich fine structures at different positions. We determined that ligands need to overcome free energy barriers of up to ∼ 5 kcal/mol when dissociating. On the other hand, while sliding, ligand encounter free energy barriers of up to ∼ 1.5 kcal/mol. Consequently, in general, sliding is favored over dissociation. Nevertheless, due to the roughness of the energy landscape and the non-homogeneity of the energy barrier locations, we hypothesize that variations in the geometry of the minor groove can be exploited to create dissociation points or “exit ramps”, to halt sliding. We found that the smallest free energy barriers are encountered by ligands that slide at the intermediate radii from the DNA axis, with the barriers being less than 1.5 and 0.8 kcal/mol for Na^+^ and methyl guanidinium, respectively. We also confirmed prior suggestions that DNA’s free energy landscape is highly modulated by its electrostatic potential, the latter being mostly determined by the sequence-dependent geometry of the minor groove. Additionally, we found that both ligands were only partial dehydrated inside the minor groove and that water-mediated interactions between the ligands and the DNA may play a critical role in favoring sliding over dissociation. Our study provides a general framework for characterizing the free energy landscapes surrounding DNA molecules and for making quantitative predictions of the energetics and molecular basis of different types of diffusive motions in the proximity of DNA chains.

## Materials and Methods

### Molecular dynamics simulations

All of our simulations were carried out using the LAMMPS MD software [39], the amber parmbsc0 forcefield for nucleic acids [40], the Joung and Cheatham ions parameters [41] and the TIP3P water model. Starting from a canonical B-form 20-base pair DNA oligomer, [d(CGCGAGGTTTAAACCTCGCG)]_2_, we solvated the system in a 50 × 50 × 70 Å box of water with periodic boundary conditions, that were applied throughout all the simulations. In addition, each strand of the DNA molecule was covalently liked to itself over the periodic boundaries of the system [42, 43]. This setup allowed us to suppress all deformation modes whose wavelengths were bigger than the system size. Na^+^ and Cl^−^ ions were added to compensate charge and to represent a 0.15 M concentration that mimics the physiological environment. The total number of atoms in the system was 15,000. The system was first minimized by 2000 cycles while constraining the DNA heavy atoms followed 1000 steps of unconstrained energy minimization using the steepest descent method. The system was then heated to 300K for 1 ns. This was followed by a round of MD simulations at constant pressure (NPT) for 1 ns using a Langevin piston pressure control to obtain a pressure of 1 atm for density equilibration. After density equilibration, the average size of the box was 48.4 × 48.1 × 67.1 Å. The final round of equilibrations was performed at 300K for 50 ns to ensure the equilibration of the ions. All equilibration and production runs were performed at constant volume, using the average volume measured in the NPT simulations. To maintain a constant temperature, a Langevin thermostat was applied with a damping constant of 0.2 ps^−1^. In equilibration and production runs the SHAKE algorithm was applied to constrain all bonds involving hydrogen atoms. Electrostatic interactions were modeled using the Particle Mesh Ewald (PME) method [44] and van der Waals interactions were truncated at 12 Å. The production run time-step was set to 2 fs and frames were saved every 2 ps. Prior works have shown that, for comparable systems (15,000—19,000 atoms), 50 ns are enough to equilibrate the NaCl atmosphere around the DNA molecule [1, 5]. During the equilibration, the DNA molecules remained in the B-form conformation. A second model included a charged arginine side chain analog, methyl-guanidinium, bound to DNA. This ligand was modeled using the amber ff99SB* force-field [45, 46] for the arginine residue. The initial coordinates of the arginine analog were obtained from the conserved -N terminal structure of the *Antennapedia* homeodomain (pdb access code 9ANT) [26].

### Helical coordinates

Taking advantage of the helical symmetry of the DNA molecules, we constructed a helical coordinates system (*ρ*, *ϕ*, *ξ*) that specifies the relative position of the ligands with respect to a DNA molecule. The DNA’s axis was aligned to the z-axis, such that the *ρ* and *ϕ* coordinates are equivalent in magnitude to the *r* and *θ* coordinates used in cylindrical coordinates (*r*, *θ*, *z*). *ξ* is the family of helical surfaces given by *ξ* = *z*−*pϕ*/2*π*, with *p* the pitch of the helical system (Fig. 1.A). It is also useful to define the pitch angle *α* such that tan*α* = *p*/2*πρ* (Fig. 1.A) and $\bar{p}=p/2\pi$. This coordinate system was initially introduced by Waldron [47] for applications in electromagnetic theory and takes the helix to be right-handed. In helical coordinates, at *ρ* = constant, the components of a vector **V** = (*V*_*ρ*_, *V*_*ϕ*_, *V*_*ξ*_), are expressed in the following way,

$$V_{\phi }\;=\;V_{\theta }\sec\alpha$$ (1)

$$V_{\xi }\;=\;V_{z}-V_{\theta }\;\tan\;\alpha$$ (2)

through the corresponding cylindrical components (*V*_*r*_, *V*_*θ*_, *V*_*z*_) (Fig. 1.B). It is important to note that the radial component of the vector *V*_*ρ*_ is perpendicular to *V*_*ϕ*_ and *V*_*ξ*_, but *V*_*ϕ*_ and *V*_*ξ*_ coordinates are not orthogonal. Analogously, the transformation between helical and cartesian (*x*, *y*, *z*) coordinates systems is given by:

$$\rho \;=\;\sqrt{x^{2}+y^{2}}$$ (3)

$$\phi \;=\;\tan^{-1}(y/x)$$ (4)

$$\xi \;=\;z-\bar{p}\tan^{-1}(y/x)$$ (5)

For the helical coordinate system we define a covariant basis (**e**_*ρ*_, **e**_*ϕ*_, **e**_*ξ*_) and a contravariant basis (**b** ^*ρ*^, **b** ^*ϕ*^, **b** ^*ξ*^). The scale factors are given by: *h*_*ρ*_ = 1, *h*_*ϕ*_ = *ρ*/ cos *α* and *h*_*ξ*_ = 1. The covariant basis vectors are defined as:

$$e_{\rho }\;=\;(\cos\phi ,\sin\phi ,0)$$ (6)

$$e_{\phi }\;=\;\left(-\cos\;\alpha \;\sin\;\phi ,\;\cos\;\alpha \;\cos\;\phi ,\;\sin\;\alpha \right)$$ (7)

$$e_{\xi }\;=\;(0,\;0,\;1)$$ (8)

The (non-unit) contravariant basis vectors are given by:

$$b^{\rho }\;=\;(\cos\;\phi ,\;\sin\;\phi ,\;0)$$ (9)

$$b^{\phi }\;=\;\left(-\frac{\sin\phi }{\cos\alpha },\frac{\cos\phi }{\cos\alpha },0\right)$$ (10)

$$b^{\xi }\;=\;\left(\frac{\tan\alpha \sin\phi }{\rho },-\frac{\tan\alpha \cos\phi }{\rho },1\right)$$ (11)

Following the definition of the gradient,

$$\nabla \Psi =\frac{1}{h_{i}}\frac{\partial \Psi }{\partial q^{i}}b^{i}$$ (12)

we expressed the helical coordinates gradient as,

$$\nabla \Psi =b^{\rho }\partial_{\rho }\Psi +b^{\phi }\frac{\cos\alpha }{\rho }\partial_{\phi }\Psi +b^{\xi }\partial_{\xi }\Psi$$ (13)

Note that Eq. 13 becomes the gradient in cylindrical coordinates in the limit *α* → 0. The helical coordinate system described above was implemented in LAMMPS.

### Umbrella sampling simulations

We used potential of mean force (PMF) umbrella sampling [48] techniques to characterize the free energy surface governing how counterions and charged residues move along the minor groove. To restrain the ligand’s center of mass, we introduced the potential:

$$\Psi (\rho ,\phi ,\xi )=\frac{k_{\rho }}{2}{\left(\rho -\rho_{0}\right)}^{2}+\frac{k_{\phi }}{2}{\left(\rho \phi -\rho_{0}\phi_{0}\right)}^{2}+\frac{k_{\xi }}{2}{\left(\xi -\xi_{0}\right)}^{2},$$ (14)

where *ρ*, *ϕ* and *ξ* correspond to the coordinates of the center of mass, *ρ*_0_,*ϕ*_0_ and *ξ*_0_ are the target positions and *k*_*ρ*_, *k*_*ϕ*_ and *k*_*ξ*_ the respective force constants. For this potential, the force (**F**) acting on the center of mass of the ligands were obtained by using the gradient in helical coordinates (Eq. 13), **F** = −∇Ψ. For each ligand we computed the PMF for one full turn (*ϕ* = 2*π*) around the DNA molecule, equivalent to a rotation along 10.5 base pairs. From the simulated segment, we computed the binding free-energy map for the mid-section (underlined sequence): [d(CGCGAGGTTTAAACCTCGCG)]_2_. The Na^+^ center of mass was constrained using force constants of *k*_*ρ*_ = 25 kcal/mol/Å^2^, *k*_*ϕ*_ = 2.5 kcal/mol/(rad^2^ ⋅Å^2^) and *k*_*ξ*_ = 10 kcal/mol/Å^2^. Umbrella increments were set to 0.4 for *ρ* and 0.25 rad for *ϕ*, for *ρ* ranging from 10 to 15 Å and *ϕ* ranging from 0 to 2*π*. The methyl-guanidinium center of mass was constrained using forced constants of *k*_*ρ*_ = 10 kcal/mol/Å^2^, *k*_*ϕ*_ = 1 kcal/mol/(rad^2^ ⋅Å^2^) and *k*_*ξ*_ = 5 kcal/mol/Å^2^. Umbrella increments were set to 0.4 Å for *ρ*, 0.25 rad for *ϕ* for *ρ* ranging from 8.6 to 12.8 Å and *ϕ* ranging from 0 to 2*π*. Force constants were chosen to ensure the proper overlap of the histograms (see S2 Fig.) and that energy contributions associated with the restraining potential in each of the three coordinates (*ρ*,*ϕ*,*ξ*) are comparable. For Na^+^, US production runs were 5 ns long for each window. For methyl-guanidinium the US production runs were 8 ns for each window. The 3D-PMF maps were obtained from the US simulations using the WHAM algorithm [49] extended to three dimensions to account the for the helical coordinate system. The PMF’s were calculated considering only the translational degrees of freedom of the ligand’s center of mass restrained in the helical coordinate system (*ρ*,*ϕ*,*ξ*). The rotational degrees of freedom, as well as the DNA’s and solvent’s degrees of freedom were averaged in the PMF. To test the convergence of the binding free-energy estimates, we repeated the WHAM calculations using only fraction of the US trajectories (S3 Fig.). The free-energy errors were estimated as integrated standard deviations of the mean using the bootstrap algorithm [50]. In each case we generated 2000 bootstraps. For visualization purposes, the 3D-PMF’s were projected into 2D-PMF’s by, at every angular position, obtaining the free energies to a “ribbon” passing through the middle of the minor groove’s solvent accessible volume (S1 Fig.). It is important to note that the helical coordinate system is not periodic. This is, for a probe with coordinates (*ρ*,*ϕ*,*ξ*), after a 2*π* rotation, the coordinates are (*ρ*,*ϕ*,*ξ* + *p*).

### Analysis of the trajectories

The geometry of the DNA molecule was analyzed using the program Curves [51], which allowed us to obtain the width of the minor groove along the studied turn. The electrostatic potential inside the minor groove was determined from the solutions of the non-linear Poisson-Boltzmann equation using the Adaptive Poisson-Boltzmann (APBS) software [52] at 0.15 M salt concentration. The atomic partial charges and radii were obtain from the Amber force field [40]. A 129 × 129 × 193 grid was used, with a grid spacing of 1.5 Å. A dielectric value of *ϵ* = 2.00 was assigned to the interior of the DNA molecule (calculate with a 1.4 Å probe sphere), whereas a dielectric value of *ϵ* = 78.54 was assigned to the solvent. Boundary condition values were determined using the Debye-Hückel approximation. To compute the correlation between the binding free-energy and the electrostatic potential we first used interpolation [53] to build a grid with a 0.13 Å spacing, equivalent to the one from the free-energy maps. The Pearson’s correlation coefficient was computed between the maps with equivalent spacing.

We identified all of the methyl-guanidinium and DNA’s minor groove hydrogen bond donors and acceptors and recorded all hydrogen bonds formed within every 2 picosecond frame along each trajectory. A geometric definition of a hydrogen bond was used: two heavy atoms are considered to be bonded if (1) their donor-acceptor distance is less than 3.5 Å and (2) the acceptor-donor-hydrogen angle is less than 60^*o*^. Additionally, we computed the radial distribution function (*g*(*r*)) between the Na^+^ ions and the oxygen atoms of the water molecules as well as the *g*(*r*) between the polar head of the methyl-guanidinum (defined as the center of mass of the NE, CZ, NH1, and NH2 atoms) and the oxygen atoms of the water molecules. For Na^+^ we computed the *g*(*r*) of cations localized to the minor groove via the umbrella potentials and for cations in the bulk in all trajectories. The hydration number of these ligands was determined by integrating, over volume, the water molecules in the first hydration shell, which is equivalent to integrating the first peak in the *g*(*r*).

## Supporting Information

S1 Fig Building the Potential of Mean Force (PMF) maps. A) The surface of the DNA is shown in blue and the region explored during the Umbrella Sampling (US) simulations, which corresponds to one helical turn, in orange. The distance between the initial and final sampling state corresponds to the pitch (p). B) Top view of the sampled region (orange). The DNA in shown in the cartoon representation (blue). C) 2D-PMF maps were built as a “ribbon” passing through the middle of the minor groove’s solvent accessible volume (orange volume in panels A and B). The red cross correspond to the methyl-guanidinium initial center of mass position.
(EPS)

S2 Fig Helical coordinates biasing potentials. A) Coordinates of a Na^+^ in a sample US trajectory. Grey lines correspond to the sampled positions (*ρ*,*ϕ*,*ξ*) and red horizontal lines to the targets (*ρ*_0_,*ϕ*_0_,*ξ*_0_). B) US overlapping histograms for the sampled *ρ* and *ϕ* coordinates. Different colors represent different radial positions.For visualization purposes we only included half the turn (*π*).
(EPS)

S3 Fig Convergence of the free-energy maps for methyl-guanidinium. A) Free energy maps obtained considering the full US trajectories data set (100%) and data sets with a fraction of the trajectories (95%, 85%, 75%, 50% and 25%). In all cases we considered the first X% of the trajectories. B) Distribution of the free energy differences between the map obtained from the full data set and the maps obtained from the data sets with a fraction of the trajectories. We observe that for data sets where the trajectories are longer than 85% of the total sampling, the differences in the free energy maps are negligible. Similar results were obtained from the Na^+^ simulations.
(TIF)

S4 Fig Steric class between DNA and Na^+^. Example from a US trajectory for which we identified a high energy barrier.
(EPS)

S5 Fig Example of the minor groove width measurements. Top left panel shows an example of the measured minor groove width. Panels A, B and C are examples of medium, wide and narrow minor grooves as shown in the top left plot. Bases highlighted in grey represent the regions where the width was measured.
(EPS)
