# Supplementary figures and images for: DNA Exit Ramps Are Revealed in the Binding Landscapes Obtained from Simulations in Helical Coordinates
Source: PLoS Comput Biol. 2015 Feb 12;11(2):e1003980. doi: 10.1371/journal.pcbi.1003980 (PMC4326129; doi:10.1371/journal.pcbi.1003980)

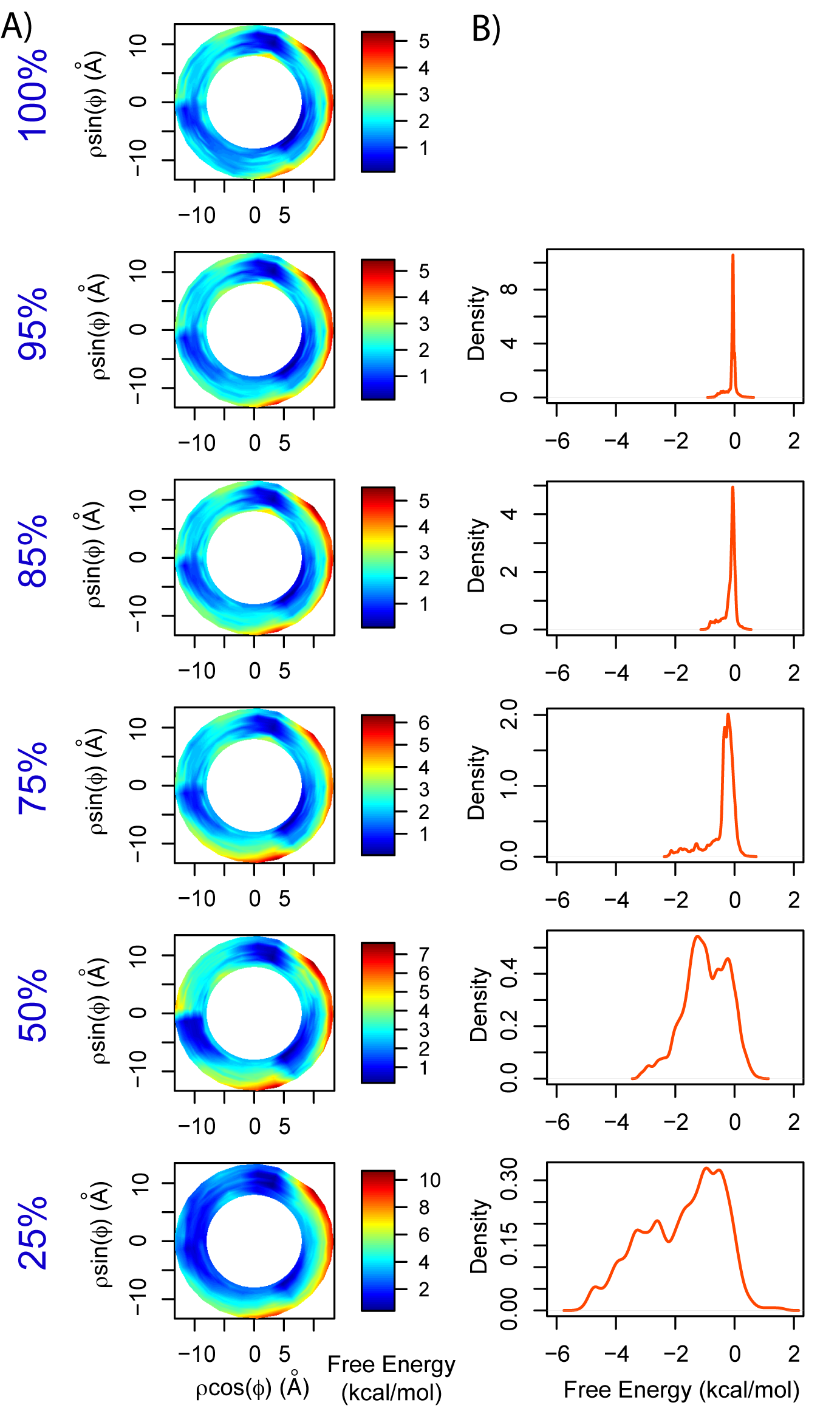

Supplement: S3 Fig — A) Free energy maps obtained considering the full US trajectories data set (100%) and data sets with a fraction of the trajectories (95%, 85%, 75%, 50% and 25%). In all cases we considered the first X% of the trajectories. B) Distribution of the free energy differences between the map obtained from the full data set and the maps obtained from the data sets with a fraction of the trajectories. We observe that for data sets where the trajectories are longer than 85% of the total sampling, the differences in the free energy maps are negligible. Similar results were obtained from the Na+ simulations. (TIF) [file pcbi.1003980.s003.tif]
